# Genetic Signatures of Competitive Performance in Burmese Gamecocks: A Transcriptomic Analysis

**DOI:** 10.3390/biology14081066

**Published:** 2025-08-16

**Authors:** Supawadee Piratae, Chanistha Yamtubtim, Thanitaporn Nonsri, Panpanit Poomprasert, Tarid Purisotayo

**Affiliations:** 1 Faculty of Veterinary Sciences, Mahasarakham University, Maha Sarakham 44000, Thailand; supawadee.p@msu.ac.th (S.P.); tongtongkomm@gmail.com (C.Y.); n.thanitaporn@gmail.com (T.N.); panpanitp@gmail.com (P.P.); 2 Veterinary Infectious Disease Research Unit, Mahasarakham University, Maha Sarakham 44000, Thailand

**Keywords:** feather, gamecock, gene expression, muscle development, neural pathways, RNA sequencing, transcriptomics

## Abstract

Understanding the genetic basis of high-performance animals is vital for biological insight and breeding. This study aimed to identify genetic factors distinguishing champion gamecocks (*Gallus gallus domesticus*) from less successful ones, representing the first study to link transcriptomic profiles directly to competitive outcomes. Using RNA sequencing on non-invasive feather samples, we compared gene expression between high-performing (≥80% win rate) and low-performing (≤20% win rate) cohorts. Our analysis identified 441 differentially expressed genes. Notably, the high-performing cocks exhibited the significant upregulation of genes integral to muscle development and repair (e.g., *SYNPO2*, *POPDC2*) and enhanced neural function (*NRN1*). Conversely, several genes involved in neural development pathways, including *CNTNAP2* and *GFRA4*, were significantly downregulated in the high-performing group. These findings suggest that a rooster’s competitive success is not determined by a single factor, but by a complex interplay of superior muscular capabilities and uniquely regulated neurological pathways. The identified genes provide a set of potential biomarkers that could inform selective breeding strategies aimed at enhancing performance traits in gamecocks.

## Simple Summary

This study aimed to uncover the genetic factors that distinguish champion gamecocks from their less successful counterparts. To find out what makes a rooster a winner, we analyzed genes from roosters with high-win records (Group A) and compared them to those from roosters with low-win records (Group B), using non-invasive feather samples. Our research revealed significant differences in the activity of 441 genes between the two groups. Notably, Group A showed increased activity in genes responsible for muscle development, function, and repair, suggesting they have a superior physical capacity. These top performers also had more active genes linked to enhanced brain function and resilience. Conversely, certain genes involved in neural development pathways were less active. We conclude that a rooster’s competitive success is likely due to a combination of enhanced muscle capabilities and specific neural traits. This genetic information could be valuable for breeders to help select for these champion characteristics, although these findings should be validated in larger populations before practical application.

## 1. Introduction

People have long been fascinated by the competitive skills of roosters. This interest is part of many cultures around the world [1]. These elite animals command high value, driving a strong interest in identifying reliable genetic markers to predict competitive potential. Understanding the genetic mechanisms that contribute to combative behaviors and physical capabilities is pivotal for informed breeding strategies aimed at enhancing performance traits. Earlier research examined traits such as aggression and feather patterns in chickens. These traits are linked to how well chickens compete, as plumage can serve as a social signal related to dominance, potentially influencing agonistic encounters [1,2,3]. These studies offer important insights, while also pointing out areas that require further investigation. Delving deeper into the genetic underpinnings of these physical traits is essential, as a thorough understanding of inherited factors could shed light on competitive behaviors and potentially shape selective breeding methods.

Studies have shown a connection between genetic markers, genes, and the competitive abilities of gamecocks. Studies have found that differences in individual aggressiveness are associated with genetic differences in adrenergic receptor genes [4] and the outcomes of competition, suggesting that males exhibiting higher levels of aggression are more likely to win [5]. Aggressive behavior can be modulated by neurochemical alterations in the brain, particularly within regions associated with reward processing. The upregulation of opioid receptor genes, which are part of the neurotransmitter systems in different brain areas, has been linked to aggressive behavior in small mammals [6] and may serve as potential molecular markers of aggression in gamecocks. Genetic factors related to muscular growth and development may contribute to an individual’s competing capabilities. Similarly, genetic factors that regulate muscular growth and development are considered fundamental to an individual’s physical capabilities, with various genes and pathways having been identified as influential in gamecocks. Such genes (e.g., *MSTN*) negatively regulate skeletal muscle growth in animals. Knocking out or disrupting the genes increases muscle mass, decreases fat deposition in chickens [7], and potentially enhances ability. In addition to studies that focus on particular genes, a genome-wide association study (GWAS) identified several other genes and pathways associated with muscle growth and development in Chinese gamecocks [8]. Despite these promising findings, connecting genetic markers to physical traits and aggressive tendencies, a critical research gap remains. Previous work has established correlations between specific genes and traits thought to be important for competition, but comprehensive studies that directly link these molecular signatures to verified competitive outcomes in authentic competitive environments are notably scarce. This gap between genotype and the ultimate performance phenotype is precisely what our research aims to address.

To achieve a comprehensive and unbiased view of the molecular activities underpinning these complex traits, this study employs transcriptome analysis via the RNA sequencing (RNA-seq). The transcriptome represents the complete set of RNA transcripts in a cell at a specific moment, offering a dynamic snapshot of active gene expression. Unlike studies focusing on a few candidate genes, RNA-seq allows for the simultaneous measurement of thousands of genes, making it a powerful discovery tool for identifying novel biological pathways and genetic signatures directly associated with an observable phenotype [9], such as competitive success.

Therefore, this study was designed to bridge this gap with three primary objectives: (1) to identify differentially expressed genes (DEGs) between high-performing and low-performing Burmese gamecocks using RNA-seq analysis of non-invasive feather samples; (2) to uncover the key biological pathways and functions associated with these DEGs; and (3) to identify a set of potential genetic biomarkers that are strongly correlated with competitive success. By measuring mRNA abundance, we directly observe the functional genetic elements that correlate with performance, providing insights that could inform marker-assisted selection in breeding programs.

## 2. Materials and Methods

### 2.1. Data and Sample Collection

All experimental protocols were approved by the Institutional Animal Care and Use Committee (IACUC) of Mahasarakham University (Permit Number: IACUC-MSU-010-056/2025). To investigate differential gene expression patterns that might distinguish between high- and low-performing Burmese gamecocks (*Gallus gallus domesticus*), we selected a cohort of 12 roosters [10] aged 12–18 months (14.4 ± 2.1) and weighted 2554–2701 g (2609.5 ± 46.6). The specific ages and live weights for each of the 12 roosters used in this study are detailed in Supplementary Table S1. Roosters were acquired from a range of distinct sources to ensure that they were not genetically related. The roosters were divided into two categories: six high-performing roosters, each with a consistent winning record in regulated competitions (at least 80% success rate in a minimum of five games), and six low-performing roosters, each with a winning rate of 20% or less in at least five games. The term ‘regulated competitions’ was defined as formal matches that adhere to local standards. This provides a standardized framework for the contests, which typically includes defined weight classes to ensure opponents are of similar size and a consistent set of rules enforced by an official. However, while these husbandry and contest rules were standardized, it is important to note that other key variables inherent to real-world competition, most notably the skill level and specific characteristics of the opponents, were not systematically controlled as part of this field-based study. All roosters were maintained under consistent environmental conditions and adhered to standardized feeding and care protocols to minimize external variables influencing gene expression. Specifically, they were housed in individual cages measuring 100 × 80 cm at temperatures ranging from approximately 25 to 30 °C, with natural light cycles. The animals received commercial feed twice daily and had ad libitum access to water. Under sterile conditions, 30 feathers were collected from the ventral wing and/or back covert feathers of each rooster. The shafts of the feathers were cut, keeping only 0.5 cm of calami tips. Care was taken to ensure that the internal pulp tissue, which remains after feather development, was collected along with the keratinous calamus tip. Feathers were chosen as minimally invasive sample sources [11]. Calami tips containing feather pulp were collected, as they are a potential source of cellular material suitable for gene expression analysis. The feather tips of each individual were placed in a two ml sterile cryotube, which was immediately transferred and stored at approximately −192 °C in liquid nitrogen.

### 2.2. RNA Extraction and Quality Control

Total RNA was extracted from collected feather calami tips. Extraction was performed according to the manufacturer’s protocol (easy-spin™ Total RNA Extraction Kit, Intron Biotechnology, Gyeonggi-do, Republic of Korea). Briefly, feather tips from each individual were placed into a two ml microcentrifuge tube. The samples were ground using a plastic pestle and homogenized in 1 mL of Lysis Buffer (easy-BLUE™ reagent, Intron Biotechnology, Republic of Korea) for two hours at 4 °C. Following homogenization, the samples were vortexed vigorously until all clumps had disappeared. Subsequently, 200 µL chloroform was added (Thermo Fisher Scientific, Waltham, MA, USA). The mixture was vortexed and centrifuged at 13,000 rpm for 10 min at 4 °C. Approximately 400 µL of the upper aqueous phase containing the RNA was carefully transferred to a new tube. An equal volume (400 µL) of Binding Buffer (Intron Biotechnology, Republic of Korea) was then added and gently mixed. The upper layer of the mixture was loaded onto a nucleic acid-binding column (easy-spin™ Total RNA Extraction Kit, Intron Biotechnology, Republic of Korea) and centrifuged at 13,000 rpm for 30 s, after which the flow-through was discarded. The column was washed sequentially with 700 µL of Washing Buffer A and 700 µL of Washing Buffer B (easy-spin™ Total RNA Extraction Kit, Intron Biotechnology, Republic of Korea). It was centrifuged at 13,000 rpm for 30 s after each wash, and the flow-through was discarded. An additional centrifugation step was performed at 13,000 rpm for 1–2 min was performed to completely dry the column membrane. Finally, the RNA was eluted by adding 50 µL of Elution Buffer directly onto the membrane, incubating for 1 min at room temperature, and centrifuging at 13,000 rpm for 1 min.

RNA quality was initially screened and RNA samples were quantified and qualified using a nano spectrophotometer (Lambda Spectrophotometer, Vacutec Ltd., Roodepoort, South Africa) and 1% agarose gel electrophoresis. Samples were required to have a minimum concentration of 10 ng/µL and a 260/280 ratio ranging from 1.8 to 2.1 to satisfy the criteria for further analysis. The integrity of the RNA was initially evaluated by examining the presence of large (28S) and small (18S) ribosomal RNA bands using agarose gel electrophoresis. Samples that exhibited two separate bands at approximately 4200 and 2300 base pairs were deemed qualified samples [12]. Samples that met these quality control standards were used for library preparation and RNA sequencing. The qualified samples were subsequently submitted for library preparation and RNA sequencing at Macrogen (Seoul, Republic of Korea).

Total RNA was successfully extracted from feather calami tips collected from all 12 gamecock samples (six high-performing, Group A; six low-performing, Group B). Quantification via nano spectrophotometry revealed RNA concentrations ranging from 26.05 ng/µL to 198.2 ng/µL with an average concentration of 75.61 ng/µL. The assessment of RNA purity revealed 260/280 ratios between 1.71 and 2.03, with most samples falling within the desired range of 1.8–2.1 specified for downstream analysis. The 260/230 ratio, indicating potential residual contamination, ranged from 1.76 to 2.1. Furthermore, RNA integrity was visually confirmed for all samples using 1% agarose gel electrophoresis, which revealed two distinct bands corresponding to the 28S and 18S ribosomal RNA subunits, meeting the qualitative criteria.

### 2.3. RNA Sequencing

Prior to library preparation using the Illumina Truseq™ stranded mRNA and total RNA kit (Illumina, San Diego, CA, USA), samples that satisfied the screening criteria were quantified using ultrasensitive fluorescent nucleic acid stain (Quant-it^TM^ RiboGreen RNA Assay, Invitrogen, Carlsbad, CA, USA). The RNA integrity number (RIN) [13] was determined using a 4200 TapeStation automated electrophoresis platform (Agilent, Santa Clara, CA, USA). Samples with an RIN greater than seven and rRNA (28s:18s) ratios of more than one were deemed eligible for library preparation [14]. The specific RIN value for each sample is provided in Supplementary Table S1. Sequencing libraries were constructed according to the manufacturer’s instructions. To select messenger RNA (mRNA) from the total RNA during library preparation, poly-A selection was employed. This method utilized the polyadenylated (poly-A) tail characteristic of most eukaryotic mRNAs. The protocols briefly included the following steps: (1) removal of DNA contamination, (2) poly-A selection, (3) RNA fragmentation, (4) reverse transcription into complementary DNA (cDNA), (5) sample-specific adaptor ligation, (6) library size selection (200 to 400 base pairs, bp), and (7) library purification and enrichment. Library qualification and quantification were conducted (2100 Bioanalyzer, Agilent, CA, USA) to assess library size and concentration. Qualified libraries were sequenced using a paired-end approach in a single lane on an Illumina HiSeq 4000 platform (Macrogen, Seoul, Republic of Korea).

### 2.4. Bioinformatic Analyses

After sequencing, the initial raw reads were subjected to quality-control checks. This step involved calculating key statistics, such as the overall quality score of the reads, the total number of bases sequenced, and the total count of reads obtained. Trimmomatic version 0.39 [15] was used to clean the raw sequencing data by first removing adapter sequences (ILLUMINACLIP) and bases at the ends of reads with a Phred quality score (Q) lower than three (TRAILING). Then, a sliding window method (SLIDINGWINDOW) was employed: scanning reads with a 4-base window and trimming bases if the average quality within the window fell below a mean score of 15. Finally, reads shorter than 36 bp were discarded after these trimming steps to produce the final trimmed data for analysis. The HISAT2 tool version 2.2.1 [16] was employed to align the final trimmed cDNA sequences with the GRCg7b chicken reference genome (NCBI RefSeq assembly GCF_016699485.2), producing binary alignment map (BAM) files. This process also yielded mapping statistics, specifically the number of processed reads, the number that aligned to the reference (mapped), and the number that did not align (unmapped).

### 2.5. Differentially Expressed Gene Analysis

With the reads mapped to the reference genome, we quantified the gene expression levels to enable comparison between the two groups. First, StringTie version 2.1.4 [17] assembled known genes (-e option of StringTie) using the reference genome. Following assembly, the expression level of each gene within a sample was determined and reported as both raw read counts and normalized values, specifically fragments per kilobase of transcript per million mapped reads (FPKM) and transcripts per kilobase million (TPM) [18]. The analysis provided key metrics for the expression level per sample, calculated for both individual known transcripts and at the aggregated gene level. These metrics included the raw read count mapped to each transcript and gene, alongside normalized FPKM values, which accounted for sequencing depth and length, as well as TPM, a normalized measure useful for comparing expression proportions across samples.

Differential Gene Expression (DEG) analysis was performed using the edgeR package version 3.34.1 [19] to quantitatively assess the expression levels of genes in the two groups of gamecocks. Genes were excluded from the analysis if they showed zero read counts in any of the 12 samples. To reduce systematic bias, size factor estimation was performed on the read count data using the calcNormFactors option, which utilizes the trimmed mean of M-values (TMM) method implemented in edgeR [19]. Briefly, this method calculated log-fold changes (M-values) for genes between samples relative to a reference, trimmed genes with extreme M-values or average expression levels, and computed a weighted average of the remaining M-values to produce normalization factors that adjust for library composition differences. Next, a statistical test was performed using the normalized data. For the comparison between the two groups, statistical significance was determined using edgeR’s exact test using a dual threshold. A gene was classified as significantly differentially expressed if it met the dual criteria of an absolute fold change (∣fc∣) ≥ 2 and an exact test adjusted *p*-value of <0.05. This combined threshold approach was employed to balance the statistical significance with the biological effect size (fold change), ensuring that the selected genes exhibited changes that were both statistically reliable and potentially biologically meaningful.

A heat map was generated to visualize the results of hierarchical clustering analysis, which utilized the Euclidean distance metric and the complete linkage method, based on the significantly differentially expressed genes. This heatmap illustrates the similarity between genes and samples based on their expression levels. To illustrate the distribution of differentially expressed genes (DEGs) in terms of statistical significance and the magnitude of fold changes, a volcano plot was constructed to compare Groups A and B. The top 20 genes were identified based on the magnitude of their absolute fold-changes.

For the significant gene lists, gene-set enrichment analysis was performed based on gene ontology database using g:Profiler [20], which classified gene products based on their related biological processes, cellular components, and molecular functions. The tool conducted a statistical test to identify GO terms that occurred more frequently in the DEG list than would be expected by chance.

## 3. Results

### 3.1. RNA Sequencing and Data Preprocessing

Following library preparation, paired-end sequencing was performed on 12 RNA samples using an Illumina platform. The initial quality assessment of the raw sequencing data revealed a substantial yield across all samples. The total number of raw reads per sample ranged from approximately 61.7 million to 86.2 million, corresponding to total raw bases between 6.2 Gbp and 8.7 Gbp. The average GC content across the samples ranged between 47.8% and 51.4%. Raw read quality was high, with the percentage of bases achieving a Phred quality score of 30 (Q30) or higher ranging from 95.5% to 96.0% per sample. After preprocessing, the total number of trimmed reads per sample ranged from approximately 60.5 million to 84.1 million, with total bases ranging from 6.1 Gbp to 8.4 Gbp. The quality metrics remained high post-trimming, with Q30 percentages ranging from 97.1% to 97.3%.

### 3.2. Bioinformatic Analysis: Read Alignment to Reference Genome

The alignment process generated BAM files for subsequent analyses. Across the samples, the number of processed reads inputted into the aligner ranged from approximately 60.5 million to 84.1 million. A high percentage of these reads was successfully mapped; the number of mapped reads ranged from approximately 57.4 million to 77.7 million per sample, corresponding to mapping rates between 92.33% and 95.39%. Consequently, the number of unmapped reads ranged from approximately 3.1 million to 6.5 million per sample, representing 4.61% to 7.67% of the processed reads.

### 3.3. Differential Gene Expression Analysis Between High- and Low-Performing Cocks

**Figure 1:**
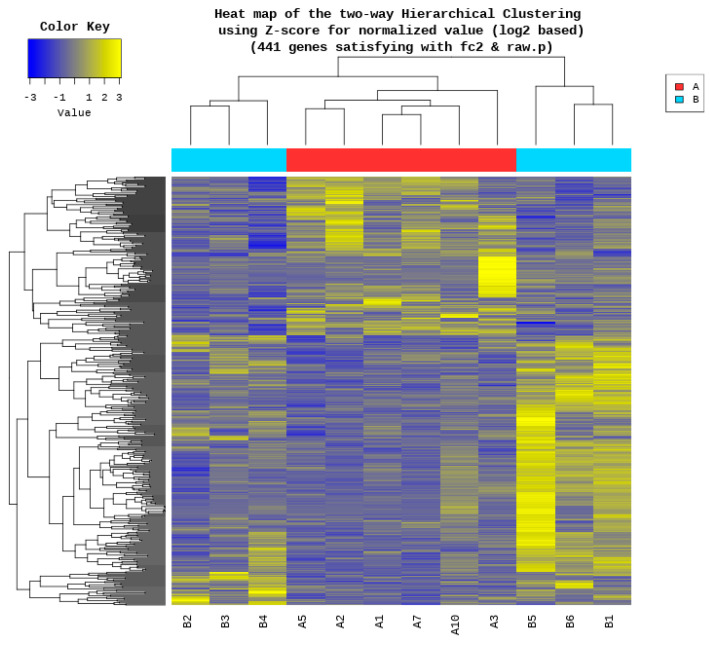
A heatmap of differentially expressed genes between high-performing (Group A) and low-performing (Group B) gamecocks. The heatmap displays the results of two-way hierarchical clustering using Euclidean distance and complete linkage for the 441 genes that were found to be significantly differentially expressed (|fold change| ≥ 2 and raw *p*-value < 0.05). Rows represent individual genes and columns represent individual samples (labeled A1, A2, A3, A5, A7, A10 for Group A, B1-B6 for Group B). Expression levels are represented by color intensity based on Z-score-normalized log2 expression values, with relative upregulation shown in yellow and downregulation shown in blue, according to the color key. The dendrograms illustrate similarity patterns among the genes (left) and samples (top). Group membership (A vs. B) is indicated by colored bars above the columns.

**Figure 2:**
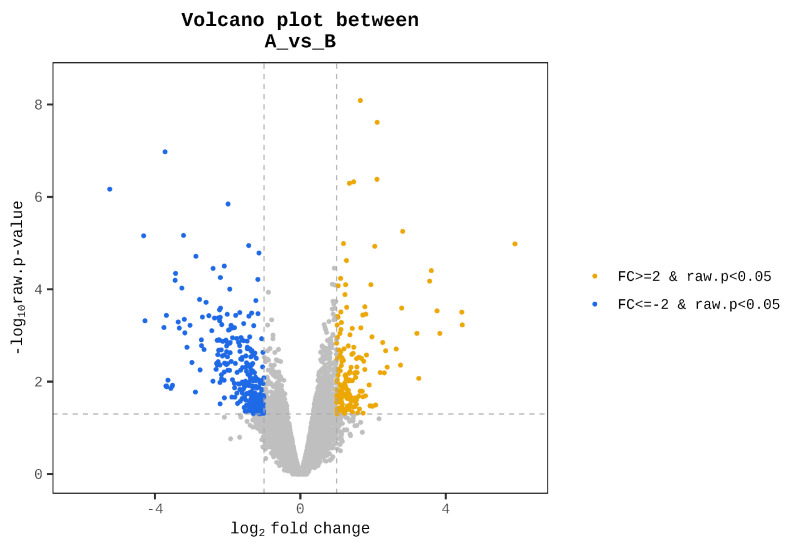
A volcano plot illustrating differentially expressed genes (DEGs) between high-performing (Group A) and low-performing (Group B) gamecocks. The *x*-axis represents the log2 fold change and the *y*-axis represents the negative log10 raw *p*-value. Genes meeting the significance thresholds of ∣log 2 FC∣ ≥ 1 (equivalent to ∣FC∣ ≥ 2) and raw *p*-value < 0.05 are highlighted. Orange points indicate significantly upregulated genes in Group A whereas blue points indicate significantly downregulated genes in Group A compared to Group B. Gray points represent genes that did not meet the significance criteria.

Genes with zero read counts in at least one of the 12 samples were excluded from further analysis, resulting in the removal of 11,176 genes from the initial set of 24,822. As a result, 13,646 genes met the quality criteria and were selected for subsequent differential expression analysis between the high- and low-performing groups. Based on 13,646 genes, 441 genes satisfied the criteria that met the dual criteria of an absolute fold change and exact test *p*-value. Among these, 163 were upregulated and 278 were downregulated in Group A compared to Group B. Please refer to the Supplementary Table S2 for all 441 genes present in the significant list. A heat map revealed a clear separation between the two groups (Figure 1). However, samples B2, B3, and B4 were more closely related to Group A samples than to the other three samples in Group B. This suggests a potential substructure within Group B, indicating the heterogeneity among its members. Significant genes were visualized in volcano plots (Figure 2). Genes located in the upper right quadrant represent significantly upregulated DEGs (163 genes), while those in the upper left quadrant represent significantly downregulated DEGs (278 genes) in Group A compared with Group B. This visualization provided a clear overview of the genes exhibiting the most substantial and statistically reliable expression differences between the two performance groups. Table 1 details the top 20 differentially expressed genes, ranked by the magnitude of change in expression levels between Groups A and B. For instance, synaptopodin 2 (*SYNPO2*) showed the highest upregulation in Group A with an approximately 60-fold increase, whereas contactin-associated protein-like 2 (*CNTNAP2*) exhibited the most significant downregulation, being almost 38 times lower in the high-performing group than in the low-performing group. Other notable genes with significant upregulation in the high-performing cocks include popeye domain containing 2 (*POPDC2*) and neuritin 1 (*NRN1*), with fold increases of approximately 12 and 7, respectively. Conversely, genes such as *GDNF* family receptor alpha 4 (*GFRA4*) and an uncharacterized locus (*LOC107055098*) were markedly downregulated, with fold decreases of approximately 20 and 13, respectively.

### 3.4. Functional Enrichment Analysis of DEGs

**Figure 3:**
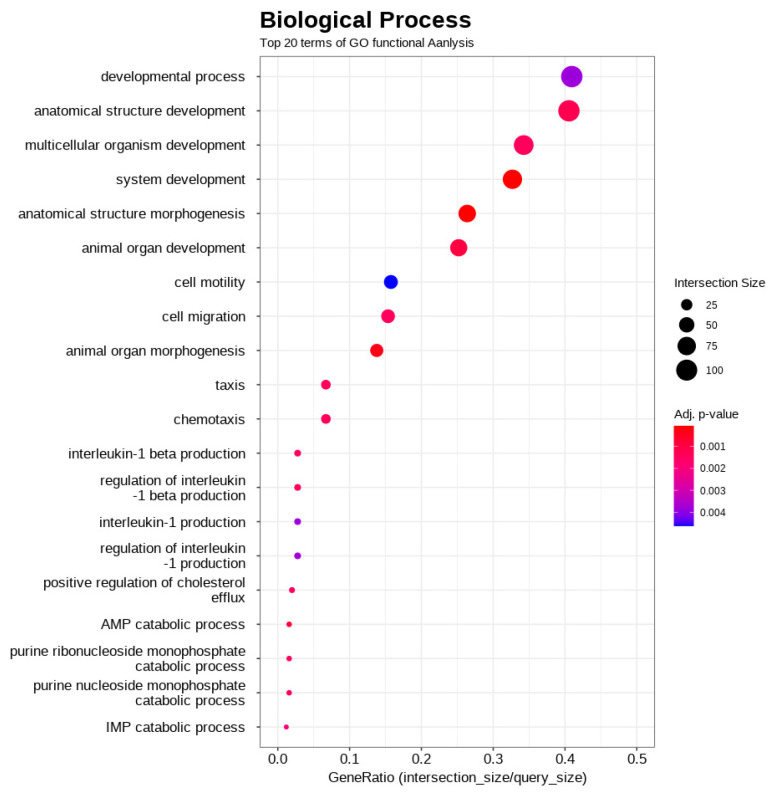
Gene ontology (GO) functional enrichment analysis of differentially expressed genes, focusing on biological processes (BP). The plot highlights the top 20 most enriched GO terms. Notably, within these biological processes, terms related to “anatomical structure development,” “system development,” and “animal organ development” animal organ development are prominent. The *x*-axis represents GeneRatio (intersection_size/query_size), indicating the proportion of genes from the input list that are annotated to a particular GO term. The size of each point corresponds to the number of genes (Intersection Size) associated with that term, and the color intensity reflects the adjusted *p*-value, indicating the statistical significance of the enrichment.

**Figure 4:**
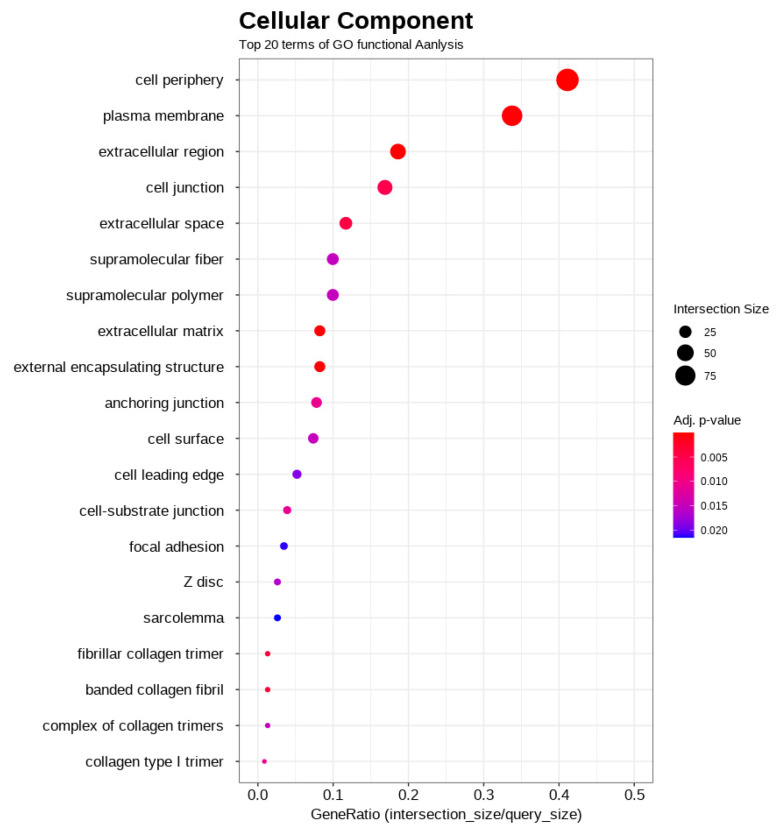
Gene ontology (GO) functional enrichment analysis of cellular components (CC), displaying the top 20 enriched terms. Significant enrichment was observed for terms associated with the “extracellular matrix” (such as “extracellular region,” and “extracellular space”) and “plasma membrane components” (including “cell periphery,” and “plasma membrane”). The x-axis, labeled “GeneRatio (intersection_size/query_size),” quantifies the proportion of differentially expressed genes associated with each GO term. The size of the points indicates the number of genes (Intersection Size) linked to a term, whereas the color represents the adjusted *p*-value, signifying the statistical significance of the enrichment.

**Figure 5:**
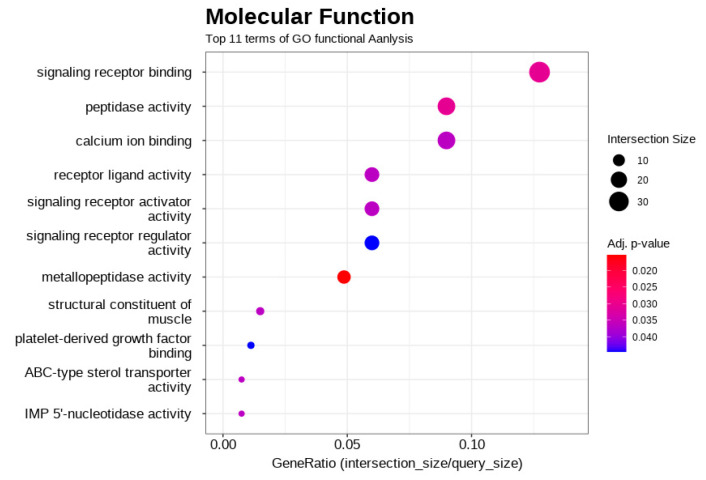
Gene ontology (GO) enrichment analysis of molecular functions (MF), highlighting the top 11 terms. Significant enrichment was observed for terms such as “peptidase activity,” “structural constituents of muscle,” and various “receptor/ligand activities” (e.g., “signaling receptor binding,” “receptor ligand activity”). The x-axis, “GeneRatio (intersection_size/query_size),” shows the proportion of differentially expressed genes annotated to each GO term. The size of each point reflects the number of genes (Intersection Size) associated with that term, and the color intensity indicates the adjusted *p*-value representing the statistical significance of the enrichment.

Functional enrichment analysis was conducted using g:Profiler on the 441 differentially expressed genes. The results revealed a significant enrichment across all three gene ontology categories. Notably, within biological processes (BP), terms related to anatomical structure development, system development, and animal organ development were prominent (Figure 3). For molecular function (MF), significant enrichment was observed for terms including peptidase activity, structural constituents of muscle, and various receptor/ligand activities (Figure 4). The most significant enrichment was found in cellular components (CC), with strong over-representation of terms associated with the extracellular matrix (e.g., collagen-containing extracellular matrix, extracellular region, extracellular space) and plasma membrane components (e.g., cell periphery, plasma membrane, membrane raft) (Figure 5). These enriched terms suggest that the differences between the high- and low-performing groups may involve developmental processes, muscle structure/function, cell signaling, and interactions within the cellular environment and extracellular matrix.

The genes contributing to these enriched GO terms revealed several DEGs potentially linked to performance traits. For instance, in terms of anatomical structure and muscle development, popeye domain-containing 2 (*POPDC2*) was significantly upregulated (approximately 12-fold) in high-performing cocks. Regarding synaptic processes, neuritin 1 (*NRN1*), which is involved in neuronal plasticity, was also upregulated nearly 7-fold. Conversely, fibrillin 2 (*FBN2*), which is involved in developmental processes and cell periphery functions, and *GFRA4*, which is crucial for neuronal function via signaling receptor activity, were notably downregulated (approximately 11-fold and 20-fold, respectively). These examples highlight specific molecular changes in muscle structure, developmental pathways, neuronal signaling components, and the cellular environment that differentiate high- and low-performing gamecocks. A complete list of DEGs is available in Supplementary Table S2, and the genes associated with specific enriched GO terms are detailed in Supplementary Table S4.

## 4. Discussion

This study directly compared genome-wide gene expression profiles between gamecocks with records of high and low degrees of competitive success. By analyzing mRNA abundance from feather calami tips in these performance groups, we identified genetic elements whose expression levels correlated with winning outcomes, linking gene activity to competitive ability. This approach moves beyond correlations with intermediate traits and offers insights into the molecular underpinnings that differentiate successful competitors. Previous studies have implicated specific gene families in aggression, such as adrenergic receptors and genes within neurotransmitter systems [4,6]. While these specific candidate genes were not among the most significantly differentially expressed in our comparison between high- and low-performing cocks, our findings did highlight the significant downregulation of genes involved in neuronal processes, such as *CNTNAP2* and *GFRA4*, in the high-performing group, suggesting that alternative or complementary neural pathways may differentiate performance levels.

In contrast, the results strongly align with the established importance of muscle development in competitive ability. Specifically, potassium calcium-activated channel subfamily M alpha 1 (*KCNMA1*), mesenchyme homeobox 2 (*MEOX2*), and *ISPD* have been previously identified as good candidates for muscle and skeletal development [7,8]. We observed the significant upregulation of several genes directly related to cardiac and skeletal muscles and function in the high-performing group, including *POPDC2*, actin alpha 1 (*ACTA1*), cyclase-associated actin cytoskeleton regulatory protein 2 (*CAP2*), cyclase-associated actin cytoskeleton regulatory protein 2 (*CSRP1*), and myosin light chain kinase (*MYLK*) (Supplementary Table S2). Among these, *POPDC2* is particularly noteworthy; its role as a myocyte-specific differentiation marker has been established during chick heart development [21], and its function in muscle regeneration is well-documented in other vertebrates [22]. Therefore, its elevated expression in high-performing cocks likely enhances their capacity for muscle repair and function during the intense physical stress of competition. This finding corroborates the expectation that enhanced muscle capabilities are probable components of success and identifies specific molecular players potentially contributing to this advantage, complementing prior work focused on genes such as (Myostatin) *MSTN* or broader GWAS findings [8].

Examining the individual genes exhibiting the most substantial differences in expression between high- and low-performing cocks provides further insights into the molecular basis of competitive success. Among the most highly upregulated genes in the high-performing group was *SYNPO2*. While its functions are primarily characterized in mammals, its role in regulating myogenesis appears to be highly conserved across vertebrate species. It is involved in muscle development through its association with synpo2 intron sense-overlapping lncRNA (*SYISL*), which regulates myogenesis in multiple species [23]. Additionally, *SYNPO2* isoforms exhibit tissue-specific expression patterns across different muscle types [24]. Thus, they play vital roles in various cellular processes across species. In humans, *SYNPO2* functions as a potential tumor suppressor, regulating autophagy and cancer progression, with high expression levels linked to a favorable prognosis in most cancers [25]. Similarly, the 60S ribosomal protein L17-like gene (*LOC107055485*), a fundamental component of the cell’s protein synthesis machinery, was also significantly upregulated in the high-performing cocks. Its upregulation in the context of the present study likely reflects a heightened capacity for ribosome biogenesis to meet high metabolic demands [26]. This increased potential for protein synthesis would be critical for supporting the continuous muscle development, maintenance, and rapid repair necessary to sustain the peak physical condition required for elite competitive performance. In addition to these findings, neuritin 1 (*NRN1*) was also upregulated by approximately 7-fold in the high-performing cohort. This is a significant finding, as evidence from mice models demonstrates that the upregulation of *NRN1* supports neuroprotection and enhances neural function [27,28,29], while its downregulation is associated with decreased neuronal survival and impaired development [27]. The increased expression of *NRN1* has been shown to improve motor function, restore synaptic integrity, and promote the growth of neurites [29]. The heightened expression of *NRN1* in high-performing cocks suggests that beyond superior muscle function, enhanced neurological adaptability, and resilience may be key contributors to competitive success. This could manifest as improved motor control, plasticity, and overall neuronal function, providing a critical advantage in competitions. Finally, pappalysin 2 (*PAPPA2*) was upregulated by over 4-fold in the high-performing group. In mammalian systems, *PAPPA2* is a metalloproteinase that enhances the local bioavailability of insulin-like growth factor (IGF) and its binding proteins (IGFBPs) [30]. Although its specific functions are not as well-characterized in avian species, the IGF signaling pathway is highly conserved across vertebrates [31]. While the downregulation of *PAPPA2* is known to cause postnatal growth retardation and skeletal abnormalities (e.g., reduced body and/or femur length) in mice models [32], its upregulation in elite cocks likely suggests the more efficient regulation of growth and tissue maintenance, further contributing to the superior physical attributes required for competitive success.

The significant downregulation of genes associated with nervous system development and neuronal function, particularly *CNTNAP2* and *GFRA4*, was observed in the high-performing group. The *CNTNAP2* gene plays a vital role in the development and functioning of the brain in animals. It is primarily responsible for regulating the development [33], structure, and activity of inhibitory neurons [34], ensuring the proper functioning of neural networks, and influencing behaviors associated with autism spectrum disorder-like (ASD-like) behaviors, epilepsy, and sensory processing [35]. The *GFRA4* gene encodes a receptor that plays a vital role in essential developmental and cellular processes in animals, particularly within the nervous and endocrine systems. *GFRA4* is crucial for the development, proliferation, and survival of specific nerve and endocrine cells [36], and its malfunction is associated with diseases [37]. Our finding highlights the potential differences in neural pathway activity and developmental timing in these birds. Interestingly, while the present study identified the downregulation of these specific neural genes, other studies have implicated different neural development genes in performance-related traits. For instance, Zhou et al. [38] identified eleven other genes involved in nervous system development that were upregulated in their study, including cadherin 18 (*CDH18*), *SLIT* and NTRK-like family members (*SLITRK1*, *SLITRK6*), N-Deacetylase and N-Sulfotransferase 3 (*NDST3*), ATP23 metallopeptidase and ATP synthase assembly factor homolog (*ATP23*), leucine-rich repeats and immunoglobulin-like domains 3 (*LRIG3*), interleukin 1 receptor accessory protein-like 1 (*IL1RAPL1*), glutamate decarboxylase-like 1 (*GADL1*), chromosome 5 open reading frame 22 (*C5orf22*), UDP glycosyltransferase 8 (*UGT8*), and WNT1-inducible signaling pathway protein 1 (*WISP1*). While downregulation might seem counterintuitive for enhanced performance [39], it could reflect modifications in neural circuitry or signaling pathways that modulate aggression, stress response, or sensory processing in a manner that is advantageous during combat. Given its role in synaptic function, the observed downregulation of *CNTNAP2* in the high-performance group might influence behavioral responses critical for success in competitions, potentially affecting aggression or social interaction dynamics. The importance of *CNTNAP2* in shaping complex behaviors has been underscored by studies in other species. For example, rats lacking the *CNTNAP2* gene exhibit behavioral changes analogous to those seen in human autism spectrum disorder [40]. The observed downregulation of genes implicated in neural connectivity (such as *CNTNAP2* and *GFRA4*) warrants further consideration. While it is tempting to hypothesize that this downregulation reflects advantageous modifications in neural circuitry related to aggression or stress response, this interpretation must be approached with caution. Several alternative explanations must be considered. First, the expression profiles were derived from feather follicles, a non-invasive but tissue-specific source. The activity of *CNTNAP2* and *GFRA4* in feathers may not be a direct proxy for their expression in the central nervous system. Second, the observed differences could be an effect of developmental timing rather than a direct correlate of adult competitive ability. The downregulation in the high-performing group might indicate an altered, perhaps accelerated, developmental trajectory where certain neurodevelopmental pathways are concluded earlier compared to the low-performing group. Although the decreased expression of such genes is known to occur with aging in some species [41], this explanation was unlikely in our study, as the animals were relatively young adults (12–18 months old). With these caveats in mind, the prominence of these neuronal genes among the top DEGs is notable. It suggests that, in addition to superior muscle physiology, the neurological profile of elite gamecocks is distinct. Future studies, ideally involving longitudinal sampling and analysis of neural tissues, are needed to disentangle these possibilities and confirm whether these expression patterns are a cause or a consequence of elite performance.

The gene ontology (GO) enrichment analysis of the 441 differentially expressed genes provides a broader biological context, revealing several key themes that differentiate the high- and low-performing gamecocks. Rather than pointing to a single pathway, the results suggest a coordinated transcriptomic signature related to developmental timing, structural remodeling, and cellular signaling (Supplementary Table S3). The most prominent theme was a difference in developmental and maturation processes. A significant cluster of enriched terms in the biological process category, including ‘anatomical structure morphogenesis’ (GO:0009653), ‘system development’ (GO:0048731), and ‘animal organ development’ (GO:0048513), points to this. Intriguingly, the examination of the associated genes, including key developmental regulators such as *RUNX2* and *PRRX1*, revealed lower expression levels in Group A than in Group B. This pattern suggests that developmental pathways are less active in Group A, perhaps reflecting earlier maturation or completion of certain developmental stages compared to Group B. This concept of gene downregulation being linked to accelerated growth has been observed in other species [42,43]. For example, the rapidly growing larvae of the Pacific oyster (*Crassostrea gigas*) showed the downregulation of genes encoding ribosomal proteins [43]. This highlights that the observed lower expression of developmental genes in Group A could signify an altered developmental trajectory, or a state of earlier maturation compared to Group B, representing a significant outcome of this functional analysis.

A second key theme involves the remodeling of cellular and extracellular structures. This is supported by the enrichment of cellular component terms like ‘cell periphery’ (GO:0071944), ‘plasma membrane’ (GO:0005886), and ‘extracellular matrix’ (GO:0031012). The differential expression of specific genes illustrates this theme vividly: for example, the upregulation of Caveolin-1 (*CAV1*), a key component of the plasma membrane involved in signaling and structural integrity [44], alongside the downregulation of collagen-type XI alpha 1 chain (*COL11A1*), an essential component for the assembly of collagen fibers in the extracellular matrix [45]. Together, these changes suggest the active modulation of the cellular environment and tissue architecture in the high-performing group.

Finally, these structural changes are linked to a third theme: the modulation of cellular signaling and response pathways. The enrichment of terms like ‘cell surface receptor signaling pathway’ (GO:0007166) and ‘response to stimulus’ (GO:0050896) highlights this. The upregulation of *CAV1* in Group A is again relevant, as it is a critical scaffolding protein that organizes signaling molecules at the plasma membrane. Collectively, the GO analysis indicates that the superior phenotype of champion gamecocks is not just about one system, but is underpinned by fundamental biological differences in developmental state, tissue structure, and cellular communication, providing valuable avenues for future investigation.

Analysis of the gene expression profiles revealed a clear clustering pattern that, for the most part, separated the high- and low-performing gamecocks, suggesting that transcriptomic signatures correlate with competitive success. However, the most intriguing finding emerged from an exception to this pattern: hierarchical clustering (Figure 1) showed three individuals from the low-performing group (B2, B3, and B4) grouping more closely with the high-performing cohort than with their own designated group. Rather than being a simple matter of intra-group variability, this finding points to a crucial insight: a simple win/loss record is an imperfect proxy for an animal’s biological potential. The molecular similarity of these “low-performing” roosters to the champion group suggests they may possess an underlying genetic capacity for high performance that was not realized in their recorded matches. This discrepancy could be due to a multitude of factors not controlled for in a real-world competitive setting, such as the superior quality of their opponents, specific match conditions, or transient environmental and physiological states.

In essence, our gene expression data may capture an animal’s innate biological capacity more accurately than their competition history alone. This highlights the profound complexity of linking genotypes to performance phenotypes and underscores the limitations of relying on simplified outcome metrics. Ultimately, this observation showcases the power of molecular profiling to uncover subtle but significant biological variations and emphasizes that future research should aim to incorporate more granular data—such as controlled opponent matching, detailed physiological measurements, and standardized performance tests—to refine experimental groupings and identify more robust biomarkers of success.

It is crucial to recognize the study’s modest sample size (*n* = 6 per group), a pragmatic necessity due to the ethical and logistical challenges of researching high-value animals. Despite this limitation, our analysis uncovered substantial effect sizes and high statistical significance for many top differentially expressed genes, such as a ~60-fold change for *SYNPO2* and ~38-fold for *CNTNAP2*. This suggests that a strong biological signal was detected, offering a robust, albeit exploratory, foundation for future validation in larger cohorts.

It is also important to consider the dynamics of the expression patterns revealed in the present study. Since this study provides a cross-sectional snapshot of adult roosters, it cannot distinguish between innate genetic predispositions evident from a juvenile stage and adaptations acquired through training. A key consideration is the challenge of using dynamic gene expression as a ‘genetic marker’ in the same way as a static DNA sequence. The identified expression signature should therefore be viewed as a molecular phenotype reflecting the current physiological state of a champion, rather than an immutable predictor. Future longitudinal studies that track these gene expression profiles from a young age and throughout a rigorous training regimen are essential to disentangle the influence of nature versus nurture. Such research would be invaluable for determining whether these signatures can predict future success or are a consequence of achieving elite status.

A further limitation of this study is its reliance on the standard GRCg7b chicken reference genome for read alignment and gene expression analysis. As a highly specialized breed, the Burmese gamecock is expected to possess significant genetic divergence from the commercial chicken breeds that form the basis of the reference genome. This use of a non-breed-specific reference introduces the potential for “reference bias,” where RNA-seq reads from gamecock-specific genes or highly divergent alleles may fail to align correctly, leading to their exclusion from the analysis or the inaccurate quantification of their expression [46,47]. Consequently, our results likely represent the expression dynamics of genes that are relatively conserved between gamecocks and the reference chicken, while potentially missing key breed-specific genes that contribute to performance. Our study provides valuable insights, the development of a high-quality, annotated gamecock-specific reference genome or a pan-genome approach [48] would be a critical step for future research to fully resolve the unique genetic architecture of this breed.

The choice of feather calami tips as the source for RNA in this study was a practical necessity, as the high value of the competing animals required a non-invasive sampling method. The use of feathers for genomic analysis is increasingly common in avian research [11]. The RNA sequencing of feather tissue has been successfully used to identify differentially expressed genes (DEGs) associated with traits like color and morphology, revealing the roles of various signaling pathways and structural genes in feather development [11]. However, it is important to consider the tissue-specific limitations of this approach when interpreting the findings. Studies have consistently shown that gene expression patterns can vary significantly depending on the source material [49,50]. For example, comparisons between cultured cell lines and their primary tissues of origin reveal that 21–32% of genes can be differentially expressed [51]. While within-sample reproducibility can be high with standardized methods, comparability across different sample types is often limited. In the present study, the gene expression profile from feather follicles is likely dominated by biological processes specific to that tissue, such as rapid cell division, keratin synthesis, and immune responses localized to the skin and feathers. Consequently, the analysis may not fully capture the gene activity occurring in other tissues more central to a gamecock’s competitive ability, such as muscle and the central nervous system. Therefore, the direct extrapolation of these expression levels to tissues like muscle or brain must be made with caution. However, the DEGs identified here provide valuable insights, particularly into the birds’ developmental and physiological state as reflected in their integument. For instance, changes in the expression of genes related to development or stress in feathers could reflect overarching physiological conditions. To build upon these findings, validation in tissues more directly related to performance (e.g., muscle biopsies or post-mortem brain tissue), coupled with robust normalization and cross-validation strategies, would help confirm the systemic role of these genes in competitive success.

## 5. Conclusions

In conclusion, our transcriptomic analysis of high- and low-performing gamecocks reveals that competitive success is a complex trait underpinned by a distinct gene expression signature. This study identified 441 differentially expressed genes that distinguish the two performance groups. The molecular profile of high-performing cocks was characterized by two main features: first, the significant upregulation of genes integral to muscle development, function, and repair, and second, a unique neurological profile marked by the differential expression of genes involved in neural plasticity and neurodevelopment. These results indicate that elite performance in gamecocks is associated with both enhanced muscular capabilities and distinct neurological pathways. The differentially expressed genes identified here provide a foundational set of candidate biomarkers. While these findings represent an important initial step, further research is required to validate these expression patterns in larger populations and in target tissues, such as skeletal muscle and the brain. Such work is necessary before these candidates could be considered for practical application in selective breeding programs. Ultimately, this study contributes to a deeper understanding of the genetic architecture underlying elite performance in this avian species.

## Figures and Tables

**Table 1:** Top 20 differentially expressed genes between high-performing (Group A) and low-performing (Group B) gamecocks were ranked by absolute fold change (∣fc∣), with positive values indicating upregulation and negative values indicating downregulation in Group A, along with corresponding adjusted *p*-values.

Rank	Gene Symbol	Description	A/B Fold Change	Adjusted *p*-Value
1	*SYNPO2*	synaptopodin 2, transcript variant X1	|59.76|	<0.001
2	*CNTNAP2*	contactin associated protein-like 2, transcript variant X4	|−37.80|	<0.001
3	*GFRA4*	GDNF family receptor alpha 4	|−19.81|	<0.001
4	*LOC107055098*	uncharacterized LOC107055098	|−13.17|	<0.001
5	*POPDC2*	popeye domain containing 2	|12.14|	<0.05
6	*LOC107055485*	60S ribosomal protein L17-like	|11.76|	<0.001
7	*FBN2*	fibrillin 2, transcript variant X2	|−10.88|	<0.05
8	*ALDH1A2*	aldehyde dehydrogenase 1 family member A2	|−10.78|	<0.05
9	*FABP5*	fatty acid binding protein 5	|−9.57|	<0.05
10	*KCNS2*	potassium voltage-gated channel modifier subfamily S member 2, transcript variant X1	|−9.27|	<0.05
11	*AGBL1*	ATP/GTP binding protein like 1, transcript variant X5	|−7.32|	<0.05
12	*NRN1*	neuritin 1	|7.03|	<0.05
13	*LOC121106479*	arylacetamide deacetylase-like 3	|−5.28|	<0.05
14	*RIMS2*	regulating synaptic membrane exocytosis 2, transcript variant X29	|−4.59|	<0.05
15	*PAPPA2*	pappalysin 2, transcript variant X1	|4.32|	<0.001
16	*FAM19A4*	family with sequence similarity 19 member A4, C-C motif chemokine like, transcript variant X2	|4.30|	<0.05
17	*SLC24A4*	solute carrier family 24 member 4, transcript variant X2	|−4.23|	<0.05
18	*SLC6A4*	solute carrier family 6 member 4	|4.13|	<0.05
19	*SLC6A15*	solute carrier family 6 member 15, transcript variant X8	|−3.97|	<0.05
20	*CRISPLD2*	cysteine rich secretory protein LCCL domain containing 2, transcript variant X2	|−3.84|	<0.05

## Data Availability

The raw sequencing data generated and analyzed during the current study are available in the European Nucleotide Archive (ENA). The study accession number is PRJEB89392, and the individual run accessions are ERR14952846–ERR14952857.

## Supplementary Materials

The following supporting information can be downloaded at: https://www.mdpi.com/article/10.3390/biology14081066/s1, Table S1: Individual rooster characteristics, including age, weight, and RNA Integrity Number (RIN); Table S2: Complete list of the 441 differentially expressed genes (DEGs) with expression statistics; Table S3: Results of the gene ontology (GO) enrichment analysis for all DEGs; Table S4: List of differentially expressed genes associated with selected enriched gene ontology terms.
